# Directed evolution provides insight into conformational substrate sampling by SrtA

**DOI:** 10.1371/journal.pone.0184271

**Published:** 2017-08-31

**Authors:** Muna Suliman, Vishaka Santosh, Tom C. M. Seegar, Annamarie C. Dalton, Kathryn M. Schultz, Candice S. Klug, William A. Barton

**Affiliations:** 1 Department of Biochemistry and Molecular Biology, Virginia Commonwealth University, Richmond, Virginia, United States of America; 2 Department of Biophysics, Medical College of Wisconsin, Milwaukee, Wisconsin, United States of America; Griffith University, AUSTRALIA

## Abstract

The Sortase family of transpeptidases are found in numerous gram-positive bacteria and involved in divergent physiological processes including anchoring of surface proteins to the cell wall as well as pili assembly. As essential proteins, sortase enzymes have been the focus of considerable interest for the development of novel anti-microbials, however, more recently their function as unique transpeptidases has been exploited for the synthesis of novel bio-conjugates. Yet, for synthetic purposes, SrtA-mediated conjugation suffers from the enzyme’s inherently poor catalytic efficiency. Therefore, to identify SrtA variants with improved catalytic efficiency, we used directed evolution to select a catalytically enhanced SrtA enzyme. An analysis of improved SrtA variants in the context of sequence conservation, NMR and x-ray crystal structures, and kinetic data suggests a novel mechanism for catalysis involving large conformational changes that delivers substrate to the active site pocket. Indeed, using DEER-EPR spectroscopy, we reveal that upon substrate binding, SrtA undergoes a large scissors-like conformational change that simultaneously translates the sort-tag substrate to the active site in addition to repositioning key catalytic residues for esterification. A better understanding of Sortase dynamics will significantly enhance future engineering and drug discovery efforts.

## Introduction

The Sortase enzymes are a ubiquitous and highly conserved family of transpeptidases that catalyze the incorporation of cell surface proteins into the prokaryotic cell wall[1–4]. The enzymes can be classified into four sub-families, A-D, based on their sequence conservation and substrate specificity. Each sub-family appears to participate in a unique physiological pathway. For example, while SrtA family members are ubiquitous in gram-positive bacteria and are primarily involved in the coupling of surface proteins required for general physiology, SrtB, SrtC, and SrtD on the other hand, are present in many fewer species and function during the iron deprivation response (SrtB) and pilin formation, respectively[2]. Several SrtA homologues are also essential for the virulence of several pathological microbes and therefore represent a viable therapeutic target for drug development[5–9].

The *Staphylococcus aureus* Sortase A (SrtA) has been the primary focus of mechanistic and structural investigations. SrtA covalently couples surface proteins containing the carboxyl-terminal pentapeptide recognition motif, or sort-tag (LP-X-TG, where X represents any amino acid), to peptidoglycan precursors on the exterior plasma membrane. Specifically, SrtA cleaves the peptide bond between the penultimate Threonine and Glycine residue[10,11] within the C-terminal sort-tag motif, forming a thioester-enzyme intermediate[12]. The reactive thioester intermediate is resolved via nucleophilic attack by an amino terminal glycine residue on the lipid II pentaglycine-bearing peptidoglycan precursor resulting in covalent ligation between the two substrates and attachment of the protein to the cell surface[1–3].

Kinetic experiments reveal that the *Staphylococcus aureus* SrtA utilizes a ping-pong double displacement mechanism with a hydrolytic shunt that is dependent on three conserved active site residues including His120, Cys184, and Arg197[10,13,14]. In an elegant analysis, Frankel and colleagues clarified the catalytic model and demonstrated that SrtA uses a reverse-protonation mechanism in which the Cys184 thiol is deprotonated and the His120 imidazole side chain is protonated during catalysis. Under these conditions, the Cys184 thiolate attacks the carbonyl of the Thr-Gly scissile bond resulting in a tetrahedral intermediate. Upon collapse of the intermediate, the nitrogen within the Gly leaving group is protonated by His120. The free enzyme is regenerated from the thioester-enzyme intermediate following nucleophilic attack by the incoming substrate amine, or less frequently, by water. The rate of transpeptidation is an order of magnitude higher than the rate of hydrolysis[13].

Sortase enzymes are characterized by a short disordered hydrophobic amino terminus that is dispensable for activity yet necessary for targeting to the cell wall. Alternatively, the carboxyl-terminus contains a small yet highly conserved catalytic domain. The SrtA catalytic domain has been structurally characterized bound to the LPETG sorting signal by x-ray crystallography and with the thiolated LPAT sort-tag analogue by NMR spectroscopy[15,16]. The crystal structure utilized the catalytically inactive C184A mutant, which despite being inactive allowed faithful recognition of the sort-tag peptide. It should be noted, however, that the structure was determined by peptide soaking, and only one of the three monomers in the asymmetric unit has clear density for the peptide. Peptide binding orders a significant portion of the β6- β7 loop, However, it remains unclear to what extent this model recapitulates the true mode of binding. The NMR model, on the other hand, utilized wild-type SrtA covalently coupled through a disulfide bond between C184 and an amino-terminally chemically protected LPAT*, where T* is a threonine analogue with the carbonyl group replaced with–CH2-SH. Collectively, the two structures reveal that SrtA adopts an eight-stranded β-barrel fold, which appears to be conserved among other Sortase family members including SrtB and SrtC[17–21]. Surprisingly however, the NMR and x-ray structures differ significantly in the orientation of the substrate and positioning of the β6- β7 and β7- β8 loops and thus fail to clearly illustrate SrtA-substrate recognition during catalysis.

SrtA has been the focus of considerable interest for the development of novel anti-microbials. However, more recently SrtA has attracted attention for its potential use as a molecular reagent to synthesize interesting bio-conjugates[12,22–29]. Indeed, SrtA can catalyze the *in vitro* conjugation of a variety of substrates including peptides, proteins, nucleic acids, and solid supports[23]. For example, Ploegh and colleagues demonstrate its potential in fluorophore attachment for *in vivo* protein localization and imaging purposes[28,30]. In this regard, SrtA is utilized in a manner similar to the biotin ligase system that has recently received widespread success[31,32]. Yet SrtA has some clear advantages to biotin ligase, including small size of sort-tag and wide variety of chemical groups that can be covalently coupled. Nevertheless, SrtA-mediated conjugation suffers from the enzymes inherently poor catalytic efficiency. In this regard, several groups have attempted to improve SrtA activity with variable success[33–36]. Directed evolution experiments of SrtA by yeast surface display and fluorescence activated cell sorting (FACS), for instance, yielded a SrtA variant with considerably enhanced catalytic efficiency as defined by a 100-fold lower K_m_ for a sort-tagged substrate and 10-fold increased k_cat_ over the wild-type enzyme[34]. More recently the same group extended their study to the identification of SrtA orthologues able to accept the altered Srt-tag sequences L-A-X-T-G and L-P-X-S-G[37].

Using a directed evolution approach based on protein complementation, we have also identified a SrtA variant with greatly improved catalytic efficiency. The combined identification of enhanced SrtA variants by our group as well as others in the context of sequence conservation and kinetic data offers considerable insight into the discrepancies between the SrtA NMR and x-ray structures and suggests a novel mechanism for sortase activity involving large conformational changes that deliver the peptide substrate to the active site during catalysis. Distance analysis of nitroxide labeled SrtA variants using electron paramagnetic resonance (EPR) spectroscopy in the presence or absence of Sort-tag peptide confirms a highly dynamic conformational equilibrium.

## Materials and methods

### Cloning and mutagenesis

For the DHFR protein complementation assay, the murine DHFR gene (Open Biosystems) was dissected into two independent domains; F-1, 2 and F-3 between residue 108 and 109. PCR was used to attach the LPETG and GGG sequences to the N- and C-terminal DHFR fragments respectively, with complementary oligonucleotides. The C-terminal fragment (F-3) was cloned into MCS-1 of pETDuet-1 (Novagen) using sites NdeI and XhoI, while the N-terminal fragment (F-1,2) was cloned into NcoI and HinDIII sites, generating the complete pETDuet-C/N-mDHFR construct. SrtA ΔN59 was cloned using ligation independent cloning into the pRSF-2 Ek/LIC vector generating pRSF-SrtAstaphΔN59 vector (Novagen). As a positive control, full-length mDHFR was cloned into the NcoI and HinDIII site of pETDuet-1. Random mutagenesis was performed using the GeneMorph random mutagenesis kit following the manufacturers suggestions (Agilent). Site-directed mutagenesis was performed using overlap-extension PCR and Phusion (NEB) as previously described. All sequences were confirmed through standard di-deoxy sequencing chemistry (Cornell University).

### DHFR complementation and SrtA library screening

For assaying DHFR complementation, pRSF-SrtAstaphΔN59 and pETDuet-C/N-mDHFR constructs were co-transformed into BL21(DE3) cells and grown in M9 minimal media in the presence of 100 μg/ml ampicillin, 30 μg/ml kanamycin, 1mM IPTG and 10 μg/ml trimethoprim, following the protocol by Remy et al.

### Protein expression

For large-scale expression, the SrtA gene was cloned into the NdeI and XhoI sites of pET28b (Novagen) as an N-terminal histidine-tag fusion protein. Following transformation into chemically competent BL21(DE3) cells (Novagen), individual colonies were picked and grown overnight in LB media at 37°C. The following day, 10mL of overnight culture was added to 1L of ZYM-5052 in baffled flasks and grown overnight at 37°C. The cells were harvested by centrifugation at 8,000xg, resuspended in lysis buffer (50mM sodium phosphate pH8.0, 300mM KCl, 1mM PMSF) and lysed using an Avestin homogenizer (average pressure of 21,000 psi). Cellular debris was removed by a 1 hour 100,000xg centrifugation step, and the clarified supernatant was loaded onto a 5mL HiTrap chelating column charged with CoCl_2_. The column was washed extensively, and eluted with a ten-column volume linear gradient to 200mM Imidazole in 20mM Tris pH8.0, 300mM NaCl. Fractions were analyzed by SDS-PAGE, and the pooled SrtA was directly loaded onto a Superdex 200 gel filtration column equilibrated in 20mM Tris pH8.0, 150mM NaCl, 1mM DTT, 10% (v/v) glycerol. Purified SrtA was pooled and flash frozen in liquid nitrogen.

### Spin labeling and DEER spectroscopy

Purified cysteine variants of SrtA were buffer-exchanged using ultrafiltration to remove DTT prior to spin-labeling with a 40-fold molar excess of MTSL (Santa Cruz Biotech.) overnight at 4°C. Excess label was removed by buffer exchange via gel filtration into 20mM Tris pH8.0, 150mM NaCl, 10% (v/v) glycerol. Labeled SrtA was pooled and flash frozen in liquid nitrogen.

Double electron electron resonance (DEER also known as pELDOR) data were collected using a standard 4-pulse sequence [38] on a Bruker ELEXSYS 580 running at Q-band with a 10W amplifier and equipped with an EN5107D2 resonator. Samples containing 100 μM protein in fire-sealed quartz capillaries (1.1mm x 1.6 mm; VitroCom), in the absence or presence of 1 mM Abz-CLEPTGG and 20% deuterated glycerol as a cryoprotectant, were flash frozen in a dry ice and acetone mixture prior to data collection at 80K. The raw data were analyzed after phase and quadratic background correction using the LongDistances software program written by Christian Altenbach (UCLA; available at http://www.biochemistry.ucla.edu/biochem/Faculty/Hubbell/) [39]. The modulation depth values and variation may be due to differences in the number of interacting spins in the observable range upon addition of peptide, the instrumental setup, or labeling efficiency. Distance distributions were determined by fitting the corrected dipolar evolution data with the model-free algorithms included in the LongDistances program followed by error analysis, which yielded standard errors for the mean distance probabilities. The expected distances between spin label pairs in the SrtA crystal and NMR structures were predicted via the PRONOX program (http://rockscluster.hsc.usc.edu/research/software/pronox/pronox.html), which is based on an experimentally derived library of allowed rotameric configurations of the spin label side chain at each site [40].

### Kinetic analysis—EDANS/Dabcyl fluorescence assay

The SrtA substrate (Dabcyl-QALPETGEE-EDANS) was purchased from Anaspec and used essentially as described except that the reactions were carried out in 384 well plates and measured in an Infinite M1000 (Tecan) plate reader[41].

### Fluorescence polarization kinetic assay

Kinetic reaction mixtures (10μL final volume) included 50mM Tris-HCl pH 8.0, 150mM NaCl, 2mM CaCl_2_, 10mM rhodamine-QALPETGG (GenScript) peptide, and varying concentrations of GGGGDYK-biotin peptide (GenScript) ranging from 0–2.5mM final concentration. Sortase protein was added to initiate the reactions at 37°C using 5μM sortase. Aliquots (1μL) were removed from the reactions at 0, 5, 10, 20, and 30 minute time points and immediately mixed with 3μL concentrated HCl. One μL of each reaction-HCl mixture was subsequently added to 75μL Neutravidin solution in a Greiner flat black 384 well plate containing 2μM Neutravidin, 50mM Tris-HCl pH8.0, 150mM NaCl, and 2mM CaCl_2_. Florescence polarization was measured in an Infinite M1000 (Tecan) plate reader using Magellan software (Tecan). Excitation and emission wavelengths of 530nm and 580nm with a 5nm width were used, respectively. Settings included 50 flashes with 100ms settle time, gain set at 70 and 0ms lag time. A Neutravidin/buffer mixture was used for background subtraction. The 0mM biotin peptide reactions with no sortase enzyme were used as references for g-factor anisotropy calculations. Linear regression analysis was performed in Excel (Microsoft). SigmaPlot 12.5 was used for kinetic analysis and statistical evaluation.

## Results

### Directed evolution of SrtA

**Fig 1:**
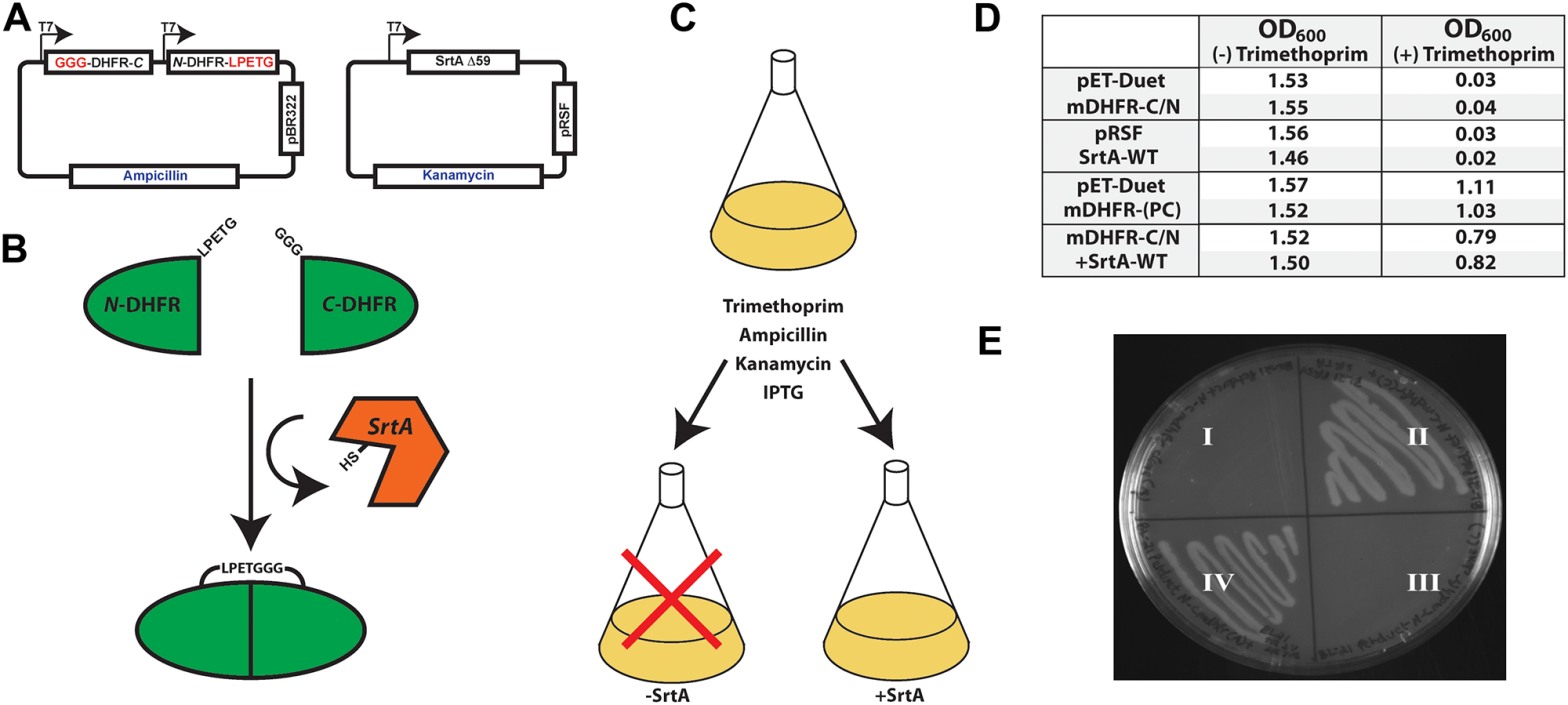
Schematic representation of the protein complementation assay utilized for directed evolution of SrtA. A) Murine DHFR is cloned into pET-Duet as two independent fragments consisting of the carboxyl-terminal region fused to three amino-terminal glycines in one open reading frame and amino terminus fused to an LPETG sort-tag in a second open reading frame. Endogenous methionine aminopeptidase (MAP) cleaves the initiating methionine to expose the terminal glycines. B) SrtA enzymatically ligates the two fragments to generate an active DHFR. Endogenous bacterial DHFR is inhibited by the prokaryotic specific trimethoprim. C) Initial culture conditions testing the requirement for SrtA in the DHFR complementation assay. D) Overnight growth of bacteria containing the various assay components in the presence and absence of trimethoprim was monitored using optical density at 600nm, OD_600_. Each trial was completed in duplicate. Cells carrying either the pET vector expressing only the split DHFR, or the pRSF vector driving expression of SrtA fail to grow in the presence of trimethoprim. Alternatively, bacteria expressing a positive control mDHFR (mDHFR-(PC)) with the internal LPETGG sequence, grow robustly in the absence of SrtA, but in the presence of trimethoprim. Similarly, bacteria expressing the split mDHFR gene along with SrtA also grow robustly following an overnight growth in trimethoprim. E) Growth of bacteria on LB-Agar plates containing Ampicillin, Kanamycin, IPTG, and trimethoprim. I) and III) BL21(DE3) cells containing pET-Duet C/N-mDHFR with empty pRSF vector, or II) and IV) BL21(DE3) cells containing pET-Duet C/N-mDHFR with pRSF-SrtA.

SrtA is a powerful molecular reagent for the synthesis of interesting bio-conjugates, yet its extensive use is hampered by poor catalytic efficiency. Therefore, to identify improved variants of SrtA, which may also aid in the further elucidation of the SrtA catalytic mechanism, we utilized the dihydrofolate reductase (DHFR) protein complementation assay originally described by Michnick et al. (Fig 1A)[42–44]. Briefly, DHFR function is essential for tetrahydrofolate synthesis and purine biosynthesis *in vivo*, and thus the DHFR protein is well conserved from bacteria to humans as demonstrated by the fact that mammalian DHFR can complement DHFR-deficient bacteria. Therefore, in the presence of trimethoprim, an inhibitor selective for bacterial DHFR, cells become dependent on an exogenous source of nucleotides, or alternatively, require ectopic expression of a mammalian DHFR homologue, which is essentially immune to trimethoprim action. For our studies, the murine DHFR gene was genetically dissected between residues 108 and 109, and expressed as amino (1–108) and carboxy-terminal (109–187) fragments fused to either the sort-tag sequence LPETG, or a tri-glycine motif (GGG), respectively. A schematic representation of the DHFR expression construct is illustrated in Fig 1A. To avoid the potential for translation of a functional DHFR via stop codon suppression, the C-terminal DHFR fragment was cloned upstream of the N-terminal fragment. In addition, it should be noted that excision of the N-terminal methionine preceding the tri-glycine motif in bacteria is carried out by methionine aminopeptidase (MAP), and thus the amino-terminus of the mature C-terminal DHFR fragment begins with a glycine *in vivo[45]*. Finally, under these conditions, SrtA activity is required for successful ligation, folding, and activity of mDHFR, the function of which is essential for bacterial growth in the presence of trimethoprim.

To verify that protein complementation by SrtA is necessary and sufficient for bacterial survival, we examined bacterial survival on agar plates and in liquid culture in the presence of trimethoprim. As can be seen in Fig 1D and 1E, *E*. *coli* expressing either the mDHFR fragments or SrtA gene alone fail to grow in minimal media in the presence of trimethoprim. Alternatively, if the mDHFR fragments and SrtA are co-expressed, the bacteria proliferate robustly in both liquid and plate culture. As a positive control, the viability of cells expressing a full-length mDHFR containing an LPETGGG linker inserted in between residues 108 and 109 was also assessed and shown to grow robustly in the presence of trimethoprim. Importantly, however, cells carrying the positive control mDHFR grow slightly better than do cells that require SrtA for survival. This suggests that SrtA activity, or mDHFR ligation, despite being well-expressed, might be limiting for bacterial survival under these conditions and leaves open the possibility that bacteria expressing SrtA variants with enhanced catalytic activity may have a selective growth advantage.

To further verify that bacterial growth is due to mDHFR fragment reassembly and is sortase-mediated, cells containing the mDHFR fragments in the presence or absence of SrtA were harvested and analyzed by SDS-PAGE and western blotting for the presence of assembled full-length mDHFR protein. As predicted, fused full-length mDHFR can only be readily identified in soluble protein extracts from cells expressing active SrtA (Fig A in S1 File) demonstrating that active mDHFR can only be reassembled from the N- and C-terminal fragments by SrtA-mediated ligation.

**Fig 2:**
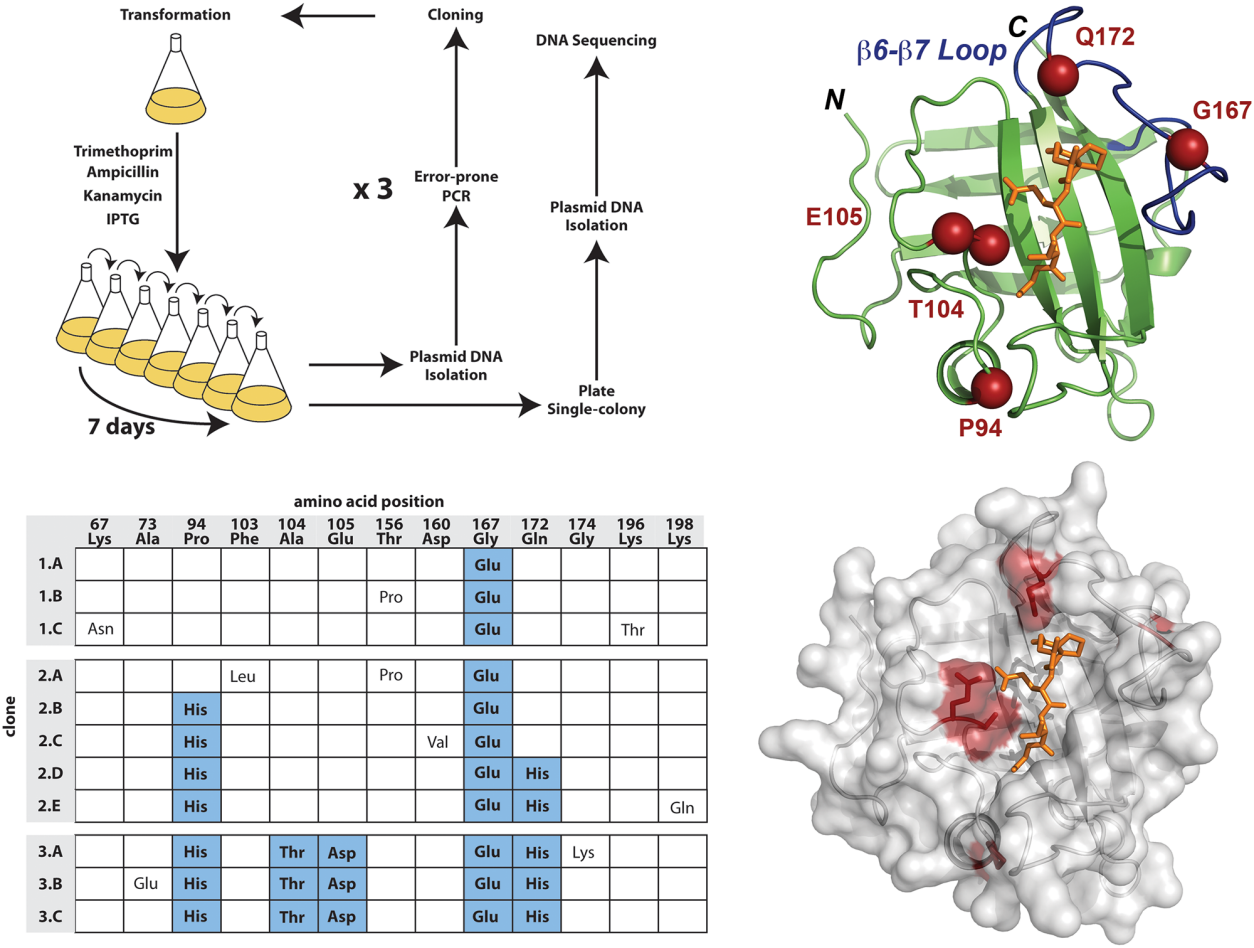
A) Basic selection protocol for directed enzyme evolution consisting of three cycles of transformation, growth, single clone picking, and sequencing. B) The frequency and location of mutations identified during rounds 1–3. Mutations that propagated through each round are highlighted in blue. C) The location of the five mutations that were isolated are shown in space-filling format in red, the β6- β7 loop is shown in blue, and the LPET peptide is shown in ball-and-stick format in orange.

For selection of catalytically enhanced enzyme variants, the core domain of the *Staphylococcus aureus* SrtA (residues 60–206) gene was amplified by error-prone PCR, cloned, and co-transformed with the mDHFR fragments to produce an initial library of approximately 2x10^7^ independent clones. Preliminary sequence analysis of several randomly selected transformants illustrated that each SrtA gene contain between one and seven mutations (data not shown). Following transformation, the library was cultured in minimal media containing IPTG for induction of protein expression, and trimethoprim to inhibit bacterial DHFR. We rationalized that the overall selective pressure for improved SrtA variants would be fairly low since it was unclear how much DHFR activity would be sufficient to permit a selective advantage over the wild-type enzyme. Therefore, over the course of a week, the culture was repeatedly grown to saturation and diluted daily to permit cells with a moderate growth advantage over time to selectively overpopulate the culture. After seven serial dilutions, plasmid DNA was isolated and re-transformed to isolate single SrtA expression plasmids for sequence analysis. Sequencing analysis revealed variants carrying between 4 and 7 mutations (Fig 2B). Under these conditions, resistance to trimethoprim by the host, or the accumulation of second-site mutations in the plasmid encoded DHFR fragments were avoided by the isolation of SrtA DNA followed by mutagenic PCR, cloning, and re-transformation into a new expression host prior to the start of subsequent rounds.

The cumulative genetic diversity observed by sequencing at the end of each round was fairly small, suggesting that the mutations that provided the greatest “fitness” to the host had overtaken the culture. Following the third and final mutagenesis and selection cycle, a total of five mutations were identified that were present among all the clones that were sequenced. However, it should be noted that due to the nature of the mutagenesis protocol (error-prone PCR), some mutations are considerably more accessible than others. Therefore, although the mutations we observe increase the overall catalytic efficiency, it remains possible that alternative amino acids at these positions might provide greater catalytic activity. Nevertheless, in support of the designed selection protocol, all five mutations (hereafter referred to as *3–5*, P94H/A104T/E105D/G167E/Q172H) localize near the sort-tag substrate as observed in the SrtA crystal and NMR structures (Fig 2C and 2D).

### Mutations increase the catalytic efficiency of acylation and transpeptidation

Previously, a manuscript by Chen et al. reported on the identification of a catalytically enhanced SrtA variant (hereafter referred to as Chen, P94R/D160N/D165N/K190E/K196T) via yeast surface display coupled with fluorescence activated cell sorting[34]. Their SrtA variant, similar to ours, contains five critical mutations necessary for enhanced activity. Interestingly, the mutations responsible for enhanced catalytic activity in their variant versus ours, share few similarities. Indeed, the only residue identified as a mutation in both studies was amino acid P94. In either case, residue P94 was mutated to a basic residue; arginine in the Chen *et al*. variant, and histidine in ours. Their remaining mutations (D160N/D165N/K190E/K196T) localize within both the β6- β7 and β7- β8 loops and point away from the ligand-binding pocket. Although the mutations in both of our SrtA variants collectively localize to the labile surface loops under investigation, it is perhaps not surprising that they cluster distinctly within the two enzyme variants. This can be explained by the observation that both directed evolution assays relied on incremental improvements. Mutations that arose first, therefore, likely influenced the evolutionary path that additional mutations would contribute to (and there is apparently more than one evolutionary path toward an improved SrtA variant). Collectively, we interpret this to indicate that structural rearrangements in *both* the β6- β7 and β7- β8 loops may contribute to the slow step in thioester formation and improvement of either one, or both, can increase acylation kinetics.

**Fig 3:**
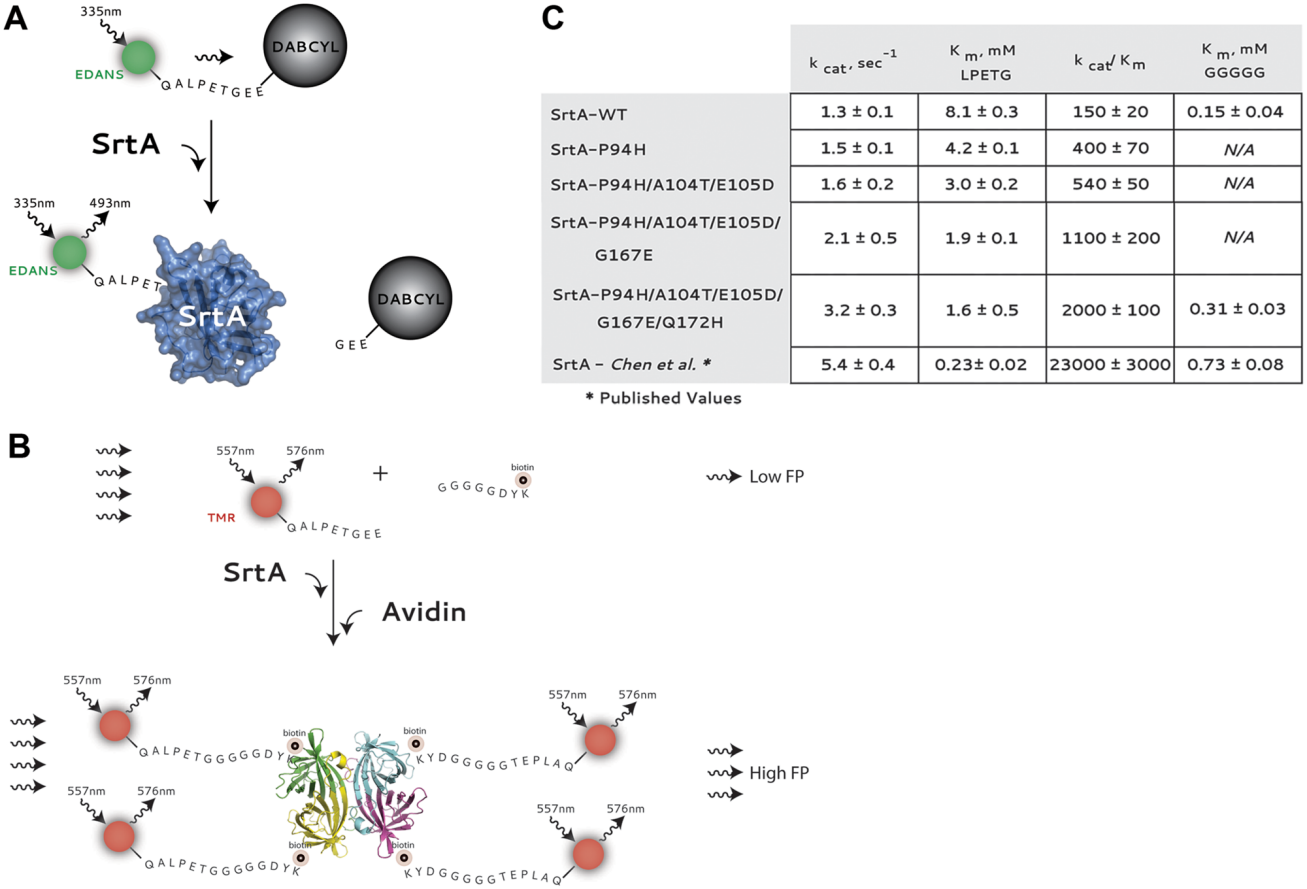
A) Schematic of the continuous fluorescence assay used to measure the initial acylation kinetics as previously described. B) Schematic of the fluorescence polarization assay used to follow the complete reaction coordinate. The peptide substrate is labeled at the amino terminus with the fluorescent label tetramethylrhodomain (TMR), which is efficiently excited at 557nm and emits at 576nm. In the absence of SrtA or secondary substrate (biotin labeled GGGGGDYK peptide), the fluorescence polarization is low. Alternatively, following the reaction of substrates in the presence of SrtA, avidin binding greatly increases the increases fluorescence polarization. C) Kinetic parameters for wild-type (WT) or SrtA variants.

Therefore, to quantitatively assess the catalytic improvements of SrtA variants, we bacterially overexpressed and purified the various enzymes to homogeneity, and evaluated their catalytic potential in a continuous fluorescence assay as previously described. Briefly, we utilized a fluorescently quenched QALPETG peptide containing the EDANS fluorophore on the amino terminus and Dabcyl quencher on the carboxyl terminus. Cleavage of the peptide by SrtA results in release of Dabcyl and de-quenching of EDANS. Fluorescence is measured in real-time on a fluorescence plate reader. Using this assay, only the formation of the thioacyl enzyme-intermediate is monitored. Nevertheless, fluorescence provides a convenient and facile means to follow the initial reaction kinetics of acylation. As a negative control, and to calculate background levels of fluorescence, we utilized the catalytically inactive SrtA-C184A variant. Using SrtA-C184A, background fluorescence at various time points was calculated and subtracted from experimental values to account for non-specific peptide cleavage or degradation (Fig E in S1 File). Kinetic parameters were first determined for the LPETG peptide substrate and can be seen in Fig 3.

Alternatively, to monitor the entire transpeptidation reaction, and obtain kinetic rate constants for the second SrtA substrate, in this case pentaglycine, we developed a novel assay based on fluorescence polarization. For these experiments, the octapeptide CALPETGG was labeled with rhodamine on the amino terminus while the octapeptide GGGGGDYK was coupled to biotin through the ε-amine within the terminal lysine residue. A schematic representation of the assay can be seen in Fig 3B. Prior to transpeptidation, the fluorescence polarization potential of the LPETG peptide is fairly low. However, as illustrated in Fig 3B, catalysis results in a larger peptide that contains both rhodamine and biotin. While fluorescence polarization is unable to distinguish between the substrate and product peptides, the polarization of the product peptide can be significantly decreased in the presence of a biotin-binding protein such as neutravidin. Thus, to evaluate the rate of transpeptidation by SrtA, we monitored the polarization of the rhodamine label following addition of SrtA. At the indicated time points, hydrochloric acid was added to quench the reaction via denaturation of SrtA. Following a brief incubation, samples were neutralized with buffer and a saturating amount of neutravidin was added to sequester biotin coupled peptide and the samples were subsequently analyzed in a fluorescence plate reader. As can be observed in Fig 3B, in the absence of SrtA, or biotin-labeled pentaglycine peptide, the polarization of rhodamine remains relatively low and constant. Importantly, this suggests that although SrtA can form a thioester intermediate with the rhodamine peptide, the total amount of enzyme-intermediate is either exceedingly small, or the overall polarization of the SrtA-peptide is not significantly different from the labeled peptide alone, or both. However, in contrast, in the presence of SrtA, a rapid change in polarization is observed (Fig E in S1 File). Shown in Fig 3C are the catalytic rate constants for the SrtA substrate, GGGGGDYK^(biotin)^. Values for the wild-type enzyme are very close to those determined previously using an HPLC assay. The Michaelis constant (K_m_) for our engineered SrtA variant, and that described by Chen et al., are roughly two and three fold higher than the corresponding value for wild-type enzyme, respectively.

### SrtA utilizes conformational sampling of the LPETG substrate

As discussed above, the NMR and x-ray structures of SrtA differ significantly in the orientation of the sort-tag substrate and positioning of the β6- β7 and β7- β8 loops. In the crystal structure, the LPETG substrate is bound to a shallow solvent-exposed crevice several angstroms from the active-site pocket. In addition, the key catalytic residues, Cys184 and His120, are improperly oriented and positioned for catalysis. Alternatively, in the NMR model the sort-tag is positioned in a deep groove created by opening of the β7- β8 loop, and residues Cys184 and His120 are properly aligned for thioester formation.

**Fig 4:**
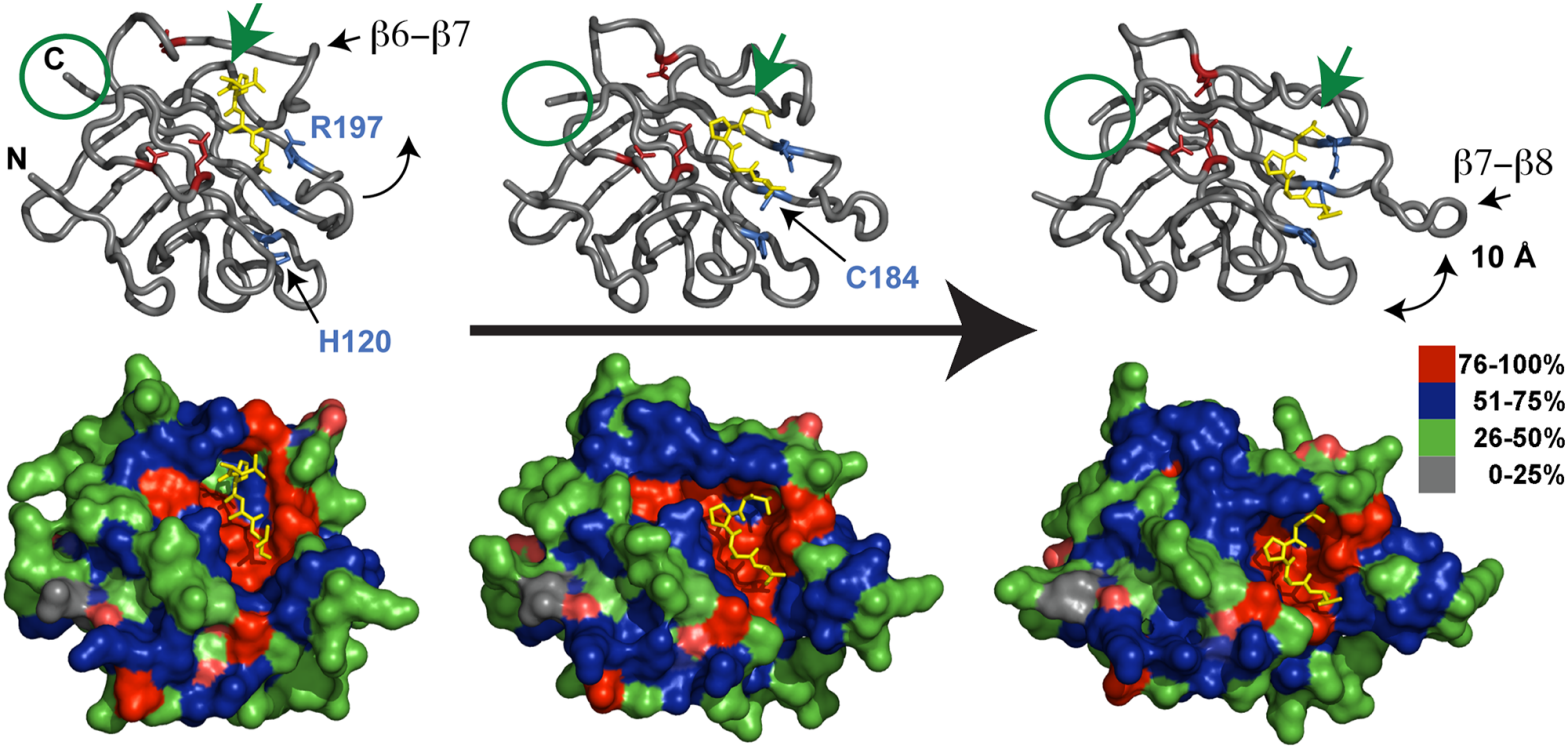
SrtA conformational changes along the reaction coordinate as depicted in the x-ray crystal structure of SrtA bound to LPETG (shown on the left) or the NMR model bound to LPAT* (shown on the right). The image in the middle is a model computationally morphed between the two structures. The top panels are in wireframe representation with the key catalytic residues in ball-and-stick format in blue, the three glutamic acids involved in Ca^2+^ chelation in red, and peptide substrate in yellow. The bottom panel is the molecular surface representation of the structure colored according to sequence conservation (among all SrtA family members). Residues that are between 76–100% conserved are shown in red, 51–75% conserved in blue, 26–50% conserved in green, and 0–25% conserved in grey. The structure on the left represents initial substrate binding. Note that the substrate sits in a highly conserved (red) pocket. Following binding, the β6- β7 loops twists and lowers/closes while the β7- β8 loop opens ~10Å in a scissor motion. Collectively, these movements guide the substrate from the top of the enzyme toward the newly formed active-site pocket in the bottom while positioning the glutamic acid residues shown in red for optimum binding of Ca^2+^ and properly orienting the residues in blue for catalysis.

It is intriguing that two independent and unique structural models were identified by complementary experimental techniques. Based on kinetic considerations of wild-type and mutant SrtA, it was originally argued that the LPETG bound substrate in the x-ray structure represents non-specific binding to the enzyme in the ground state. Alternatively, based on the localization of mutations within conformationally flexible regions that enhance catalysis, including the β6- β7 and β7- β8 loops, we postulated that SrtA undergoes a number of uncharacterized yet essential structural rearrangements following ligand binding. Specifically, we hypothesized that the x-ray crystal structure represents the initial substrate-binding event prior to catalysis, and that a large conformational change within the enzyme induces movement of the substrate deeper into the active site pocket (as shown on the left in Fig 4). For catalysis to occur, the enzyme must reorient and close the β6- β7 loop, bringing the residues within the calcium-binding motif into proper orientation for calcium binding, while also opening the active site pocket through the separation of the β7- β8 loop from the protein core. The catalytic residues Cys184 and His120 also rotate and approach one another and adopt positions appropriate for catalysis. The SrtA-substrate NMR model, despite containing an inappropriate disulfide bond between the enzyme and sort-tag substrate, roughly approximates the catalytically competent enzyme (shown on the right in Fig 4).

To more fully illustrate the dynamic events that must occur following substrate binding, we computationally morphed intermediate structures between the x-ray and NMR structures (due to space constraints we can only show one of these images—see middle panel in Fig 4). A brief examination of the different models colored according to sequence conservation reveals that the ligand-binding pocket, despite being distinct in the x-ray and NMR models, are all highly conserved (red in Fig 4), suggesting that it is very unlikely that substrate recognition is either non-specific, or induced by crystal contacts in the x-ray crystal structure. Similarly, residues within the β6- β7 and β7- β8 loop are also highly conserved (mostly blue in Fig 4) signifying that although they do not directly contribute to the initial ligand binding event, they may play an important role in catalysis.

**Fig 5:**
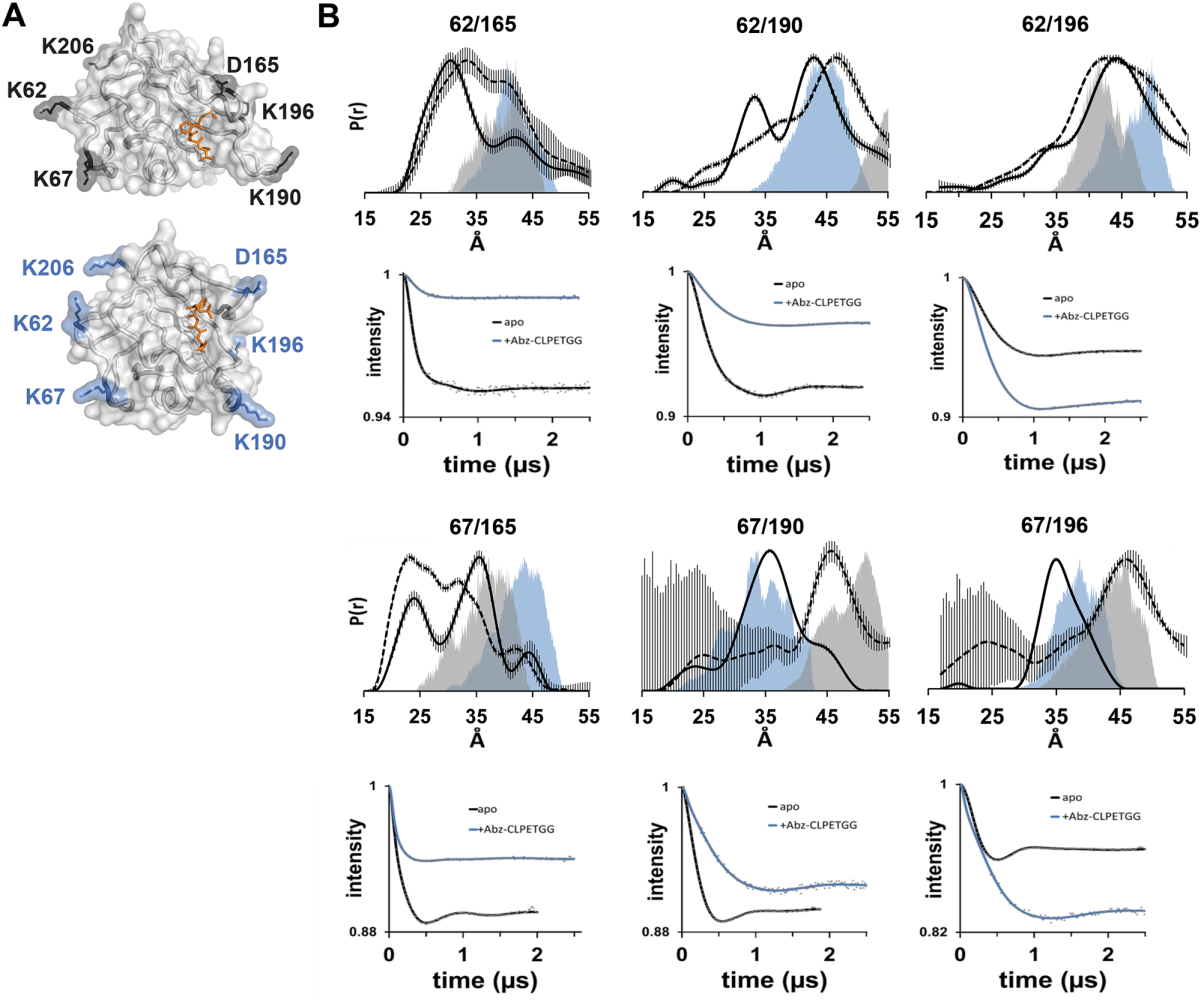
SrtA is a dynamic protein that undergoes a large conformational change upon substrate binding. A) Residues selected for mutagenesis for spin-label incorporation are labeled and their positions are shown in the NMR (top) or x-ray crystal structure (bottom) models. B) Distance distribution plots of SrtA variants with pairs of spin labels incorporated at the indicated positions in the absence (solid line) or presence of sort-tag substrate peptide (dashed line). The colored distributions represent the predicted distances obtained from PRONOX using the x-ray (blue; PDB: 1T2W) and NMR models (gray; PDB: 2KID-1). The background-corrected dipolar evolution data (gray dots) are shown for each pair of spin labeled mutant SrtA proteins (100 μM) as recorded on a Q-band Bruker ELEXSYS 580 spectrometer (Fig B in S1 File for the raw data); the black lines represent the fits to the data in the absence of substrate and the blue lines represent the fits to the data for the SrtA protein in the presence of 10x Abz-CLEPTGG.

In an effort to examine the basis for the differences between the crystal and NMR models, and potentially observe conformational changes that may occur within SrtA following substrate binding, we utilized site-directed spin-labeling and double electron electron resonance (DEER) spectroscopy to specifically follow movements in the β6- β7 and β7- β8 loops upon substrate binding. Using site-directed mutagenesis, pairs of cysteine residues were introduced into catalytically inactive, cysteine-less SrtA (C184A). Five residues (K62, K67, D165, K190, and K196) were chosen for investigation based on their ability to distinguish between the proposed conformational states of SrtA (See Fig 5A). Residues K62 and K67 are within the amino terminus and their C-alpha positions are approximately 15 Å apart in both structures. K62 and K67 are on the opposite face of SrtA from the β6- β7 and β7- β8 loops, and remain relatively constant in relation to the remaining core residues of SrtA. On the other hand, D165 is within the β6- β7 loop, and K190 and K196 are both within the β7- β8 loop. Thus, the pairs of spin labeled residues K62/D165 and K67/D165 report on the movement of the β6- β7 loop relative to the protein core and the pairs of spin labeled K62/K190, K62/K196, K67/K190, and K67/K196 report on changes of the β7- β8 loop relative to the protein core. It should be noted, however, that for simplicity during spin labeling, all our mutants have a C184A mutation that prevents catalysis. Furthermore, because of our labeling methodology, we have not made spin label variants in a C184 background for kinetic studies.

Following expression and purification, individual SrtA variants were labeled and Q-band DEER spectroscopy data were collected (Fig 5). The corresponding distance distribution plots for the background-corrected dipolar evolution data for each of the six SrtA variants can be observed in Fig 5B; the distribution plots include the predicted distance distributions obtained using the program PRONOX for the pairs of spin labels as observed in either the NMR (PDB: 2KID-1) or crystal structure (PDB: 1T2W) models. As the data highlights, the spin label motion is highly mobile, which indicates that the loops remain highly mobile even when spin labeled. However, as can be observed in Fig 5B, Figs B and C in S1 File, the experimental distances for pairs K62/K190, K62/K196 and K67/K196 appear to be most consistent with the position of the β7- β8 loop as observed in the X-ray structure model while the experimental distances from K67/D165 appear to be somewhat more consistent with the position of this loop in the NMR structure. Interestingly, the experimental distances between the spin labels attached to SrtA K62/D165 and K67/K190 in the absence of substrate do not directly support either model, though the predicted distances from the models for all pairs do overlay some part of the broad distance distributions observed. The widths of the distribution plots suggest that the spin labels at these surface exposed sites are fairly mobile; the continuous wave EPR spectra support this suggestion (Fig D in S1 File). This observation, coupled with the varying level of agreement between the three datasets, indicates SrtA is dynamic and able to stably adopt multiple conformational states.

In an effort to identify changes that occur following introduction of the sort-tag substrate, we also collected data on the same double labeled SrtA mutants in the presence of an Abz-labeled peptide substrate, CLPETGG (the Abz-group was added for quantification purposes). Interestingly, we find that the distance distributions between spin labels at K67 and K190 or K67 and K196 increases by 10 Å in the presence of the sort-tag substrate (Fig 5B and Fig C in S1 File). Similarly, the total mean distance populations between spin labels at K62 and D165 and K62 and K190 also increase, while the total mean population of distances between K67 and D165 decreases. The data also indicate that in general, there is little change between K62 and K196 upon addition of the Abz-labeled substrate peptide. Collectively, these results are consistent with the idea that the β7- β8 loop opens significantly following substrate binding to properly position the substrate and enzyme for catalysis.

## Discussion

As a key therapeutic target and focus of several enzyme-engineering efforts, it is critical to fully characterize the SrtA catalytic mechanism with particular attention paid to the identification of rate-limiting steps. Toward this goal, we evolved a SrtA variant with enhanced catalytic activity containing 5 mutations, which primarily localize to the β6- β7 and β7- β8 loops outside the direct substrate-binding pocket. Our results along with other published mutations known to enhance catalysis in the context of the discrepant crystal and NMR models of the *S*. *aureus* SrtA, suggest that SrtA may undergo substrate sampling prior to catalysis. To validate our hypothesis, we followed the conformational states sampled by SrtA prior to and following substrate binding using DEER spectroscopy. Our data demonstrate distinct conformational states of SrtA prior to and following sort-tag binding that are consistent with the crystal structure of SrtA representing the initial substrate-binding event and the NMR model consistent with the catalytic intermediate, or covalent enzyme-substrate thioester intermediate. Based on the experimental distances obtained between 67 and the β6- β7 and β7- β8 loop sites in the presence and absence of Abz-CLEPTGG, it is clear a large conformational change occurs upon substrate binding. Interestingly, the distance changes between 62 (15 Å away from 67) and the β6- β7 and β7- β8 loop sites upon substrate binding are not as large as observed using 67 as the anchor site. This could be due to 62 moving upon substrate binding, or to the combination of spin labels at these sites affects the affinity of substrate binding, or more likely it is due to the geometry of the movement of 190 and 196 with respect to 62 (e.g., a lateral movement that does not affect the distance between the pair of labels) such that there is very little change in distance. Further, an additional peptide variant (TMR-QALPETGT) and a control peptide (GGGGGGK) were also tested with the 67/196 SrtA protein and both showed no change in the mean distance indicating that SrtA does not bind to, or does not change conformation in the presence of, these peptide variants as was observed for the Abz-CLPETGG peptide (Fig C in S1 File).

Although dynamic conformational changes have been implied in several computational studies[46,47], until now direct experimental evidence for such states was lacking as was an understanding of how and why alterations to the β6- β7 and β7- β8 loops could facilitate or hinder catalysis. Indeed, our model consolidates the two discrepant structural models provided by x-ray crystallography and NMR, is consistent with conformational changes of active site residues which must occur prior to catalysis in support of the reverse-protonation catalytic mechanism proposed by Frankel and colleagues, and provides a logical explanation for SrtA mutations that enhance catalysis despite their significant distance from the active site pocket. It is tempting to speculate that such a unique mechanism of conformational sampling may reflect distinct chemical environments required for thiolate formation, which are better achieved when C184 is solvent exposed. Alternatively, recognition of the sort-tag (LPETG), proper geometric arrangement of the catalytic residues, and catalysis may all be more efficient when the enzyme is in the “closed” form. In agreement, conservation within the SrtA family appears to focus on residues involved in recognition and movement of the incoming LPETG substrate whereas the remaining parts of the molecule remain only sparingly conserved. Further supporting our conclusions, the Liu lab recently reported the identification of SrtA enzyme variants capable of utilizing altered sort tags, in this case either L-P-E-S-G or L-A-E-T-G[37]. As expected, their latest enzyme variants had mutations that primarily localized to the substrate-binding pocket and not the conformationally labile β6- β7 and β7- β8 loops.

## Supporting information

S1 File Supplemental information file including Fig A–E in S1 File. **Fig A in S1 File:** Immunoblot using anti-DHFR antibodies to demonstrate synthesis and ligation of mDHFR in the presence of SrtA. SrtA expression was induced in BL21 (DE3) cells in the presence or absence of the separate mDHFR fragments (mDHFR(C/N)), or the positive control variant of mDHFR (mDHFR(PC)). Assembled and ligated mDHFR is present in lanes 2 and 3, and migrates at the same molecular weight as the positive control. **Fig B in S1 File:** The raw dipolar evolution data (gray dots) and backgrounds (solid lines) are shown for each pair of spin labeled mutant SrtA proteins (100 μM) as recorded on a Q-band Bruker ELEXSYS 580 spectrometer. The black lines represent the backgrounds for the data in the absence of substrate and the blue lines represent the backgrounds for the data for the SrtA protein in the presence of 10x Abz-CLEPTGG. Data were analyzed using the freely available LongDistances program (http://www.biochemistry.ucla.edu/biochem/Faculty/Hubbell/). **Fig C in S1 File:** Distance distributions (top) and dipolar evolution data (bottom) for the K67/K196 spin labeled pair upon addition of 10x GGGGGGGK (tan) or TMR-QALPETGT (orange). The increase in distribution width suggests the protein becomes more flexible in the presence of these peptides, and the lack of a mean distribution shift indicates that these peptides do not induce a conformational shift in the position of the β7-β8 loop. **Fig D in S1 File:** The X-band CW EPR spectra for the indicated MAL-6 labeled single cysteine mutants of cys-less (C184S) SrtA are shown. Spectra were recorded on a Bruker ELEXSYS 500 spectrometer with a Bruker super high Q resonator at room temperature over 100 G, with field modulation of 1 G under nonsaturating conditions, and signal averaged 9 times. The spectra all indicate relatively fast motion of the spin label side chains, consistent with their locations on the exterior of the protein surface. The addition of 30% Ficoll to K62 or K206 SrtA (gray lines) did not slow the motion of the labels, confirming that the spectra are reporting on side chain mobility and not on protein tumbling. **Fig E in S1 File:** Raw kinetic data for the kinetic experiments in Fig 3.
(DOCX)
