# Supplementary material for: Directed evolution provides insight into conformational substrate sampling by SrtA
Source: PLoS One. 2017 Aug 31;12(8):e0184271. doi: 10.1371/journal.pone.0184271 (PMC5578623; doi:10.1371/journal.pone.0184271)
Supplement: S1 File — Fig A in S1 File: Immunoblot using anti-DHFR antibodies to demonstrate synthesis and ligation of mDHFR in the presence of SrtA. SrtA expression was induced in BL21 (DE3) cells in the presence or absence of the separate mDHFR fragments (mDHFR(C/N)), or the positive control variant of mDHFR (mDHFR(PC)). Assembled and ligated mDHFR is present in lanes 2 and 3, and migrates at the same molecular weight as the positive control. Fig B in S1 File: The raw dipolar evolution data (gray dots) and backgrounds (solid lines) are shown for each pair of spin labeled mutant SrtA proteins (100 μM) as recorded on a Q-band Bruker ELEXSYS 580 spectrometer. The black lines represent the backgrounds for the data in the absence of substrate and the blue lines represent the backgrounds for the data for the SrtA protein in the presence of 10x Abz-CLEPTGG. Data were analyzed using the freely available LongDistances program (http://www.biochemistry.ucla.edu/biochem/Faculty/Hubbell/). Fig C in S1 File: Distance distributions (top) and dipolar evolution data (bottom) for the K67/K196 spin labeled pair upon addition of 10x GGGGGGGK (tan) or TMR-QALPETGT (orange). The increase in distribution width suggests the protein becomes more flexible in the presence of these peptides, and the lack of a mean distribution shift indicates that these peptides do not induce a conformational shift in the position of the β7-β8 loop. Fig D in S1 File: The X-band CW EPR spectra for the indicated MAL-6 labeled single cysteine mutants of cys-less (C184S) SrtA are shown. Spectra were recorded on a Bruker ELEXSYS 500 spectrometer with a Bruker super high Q resonator at room temperature over 100 G, with field modulation of 1 G under nonsaturating conditions, and signal averaged 9 times. The spectra all indicate relatively fast motion of the spin label side chains, consistent with their locations on the exterior of the protein surface. The addition of 30% Ficoll to K62 or K206 SrtA (gray lines) did not slow the motion of [file pone.0184271.s001.docx]

**Fig A in S1 File**


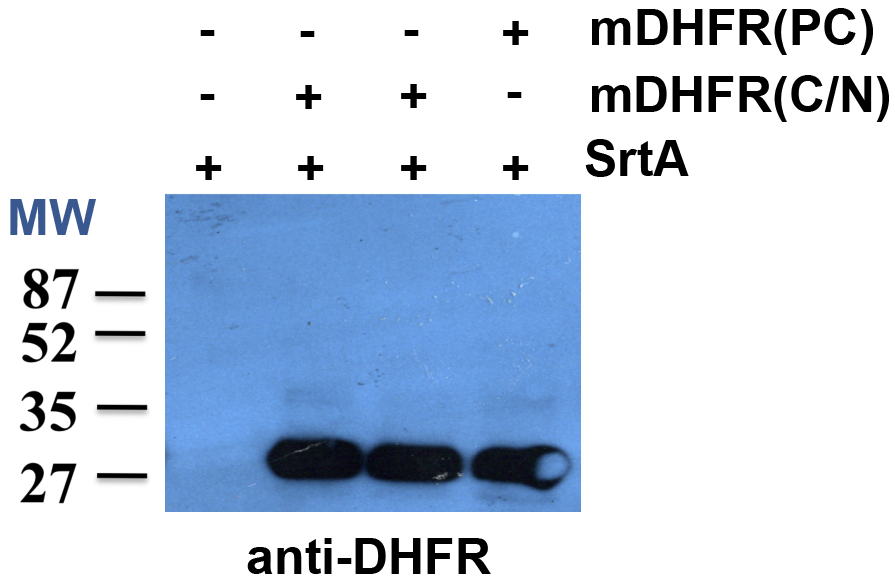


**Fig B in S1 File**


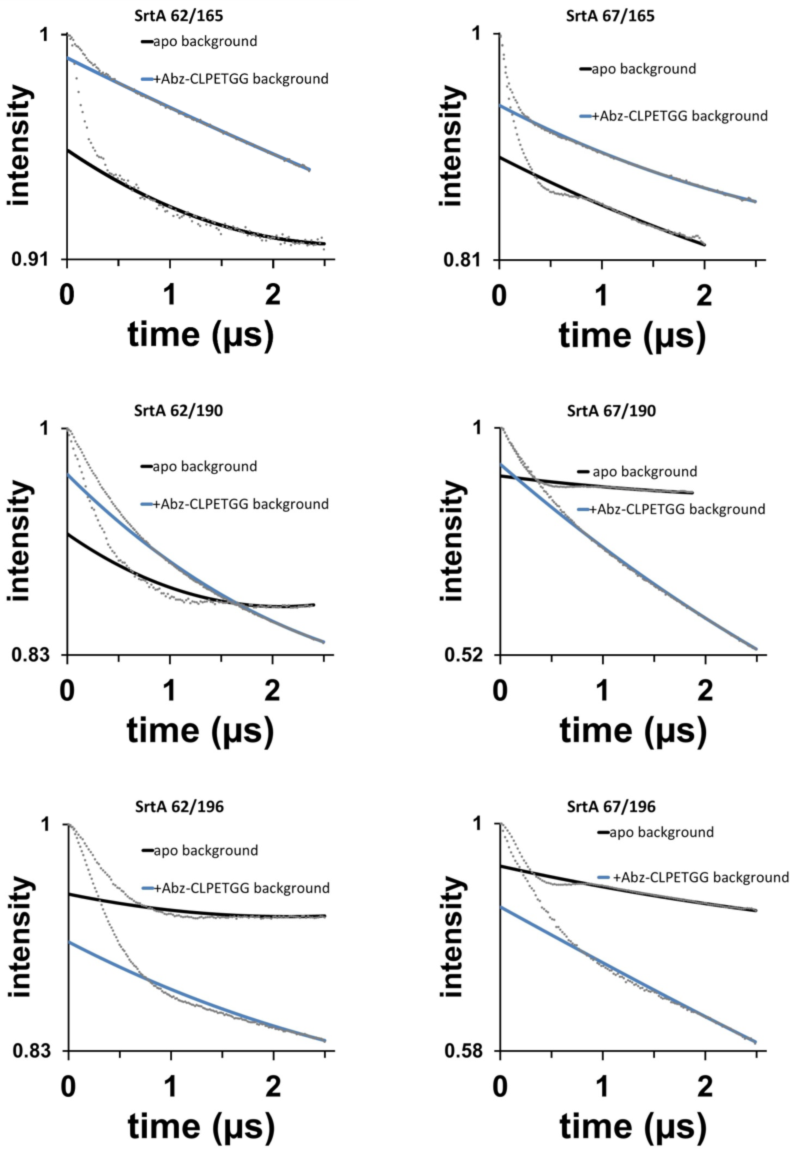


**Fig C in S1 File**

**
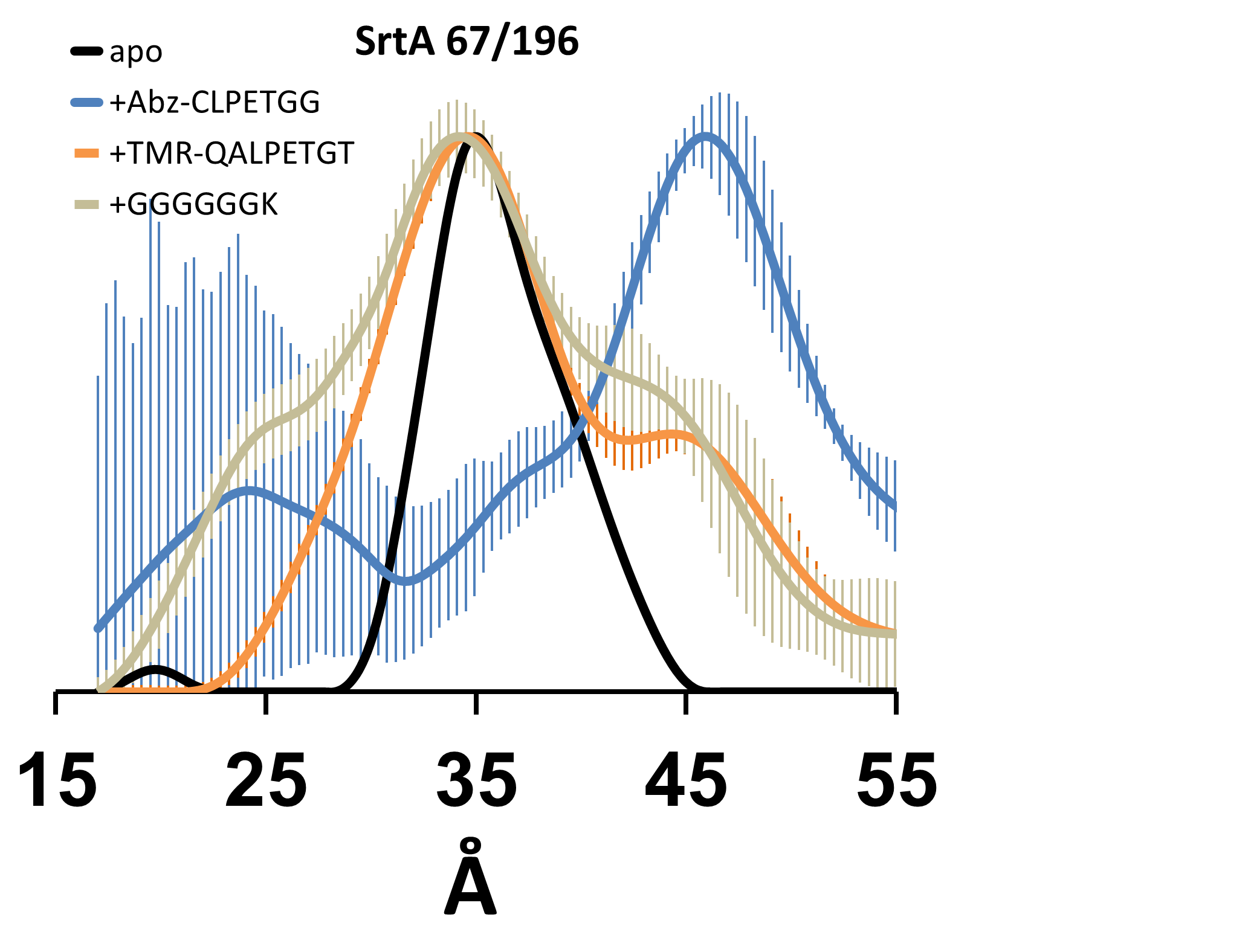
**

**
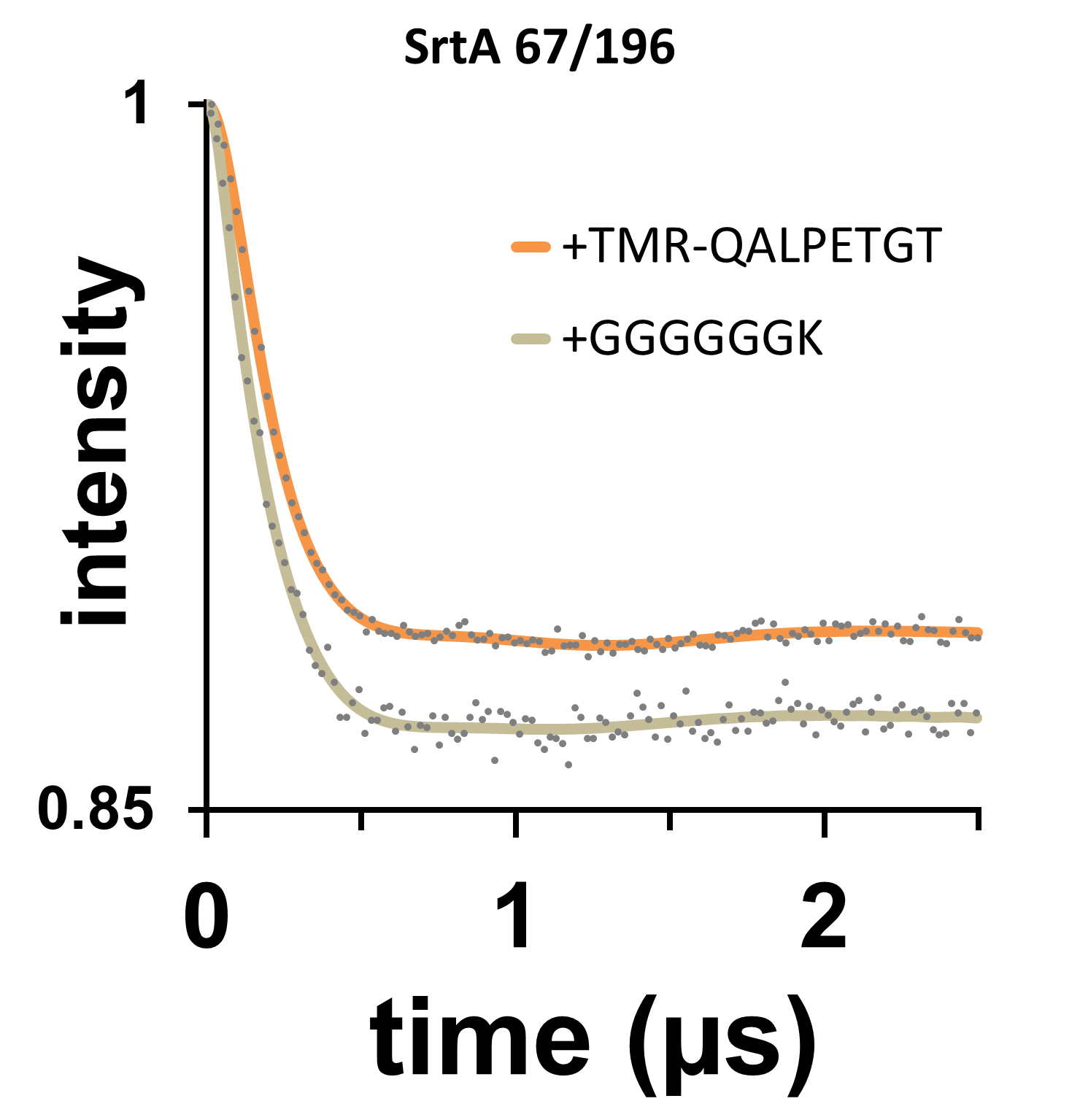

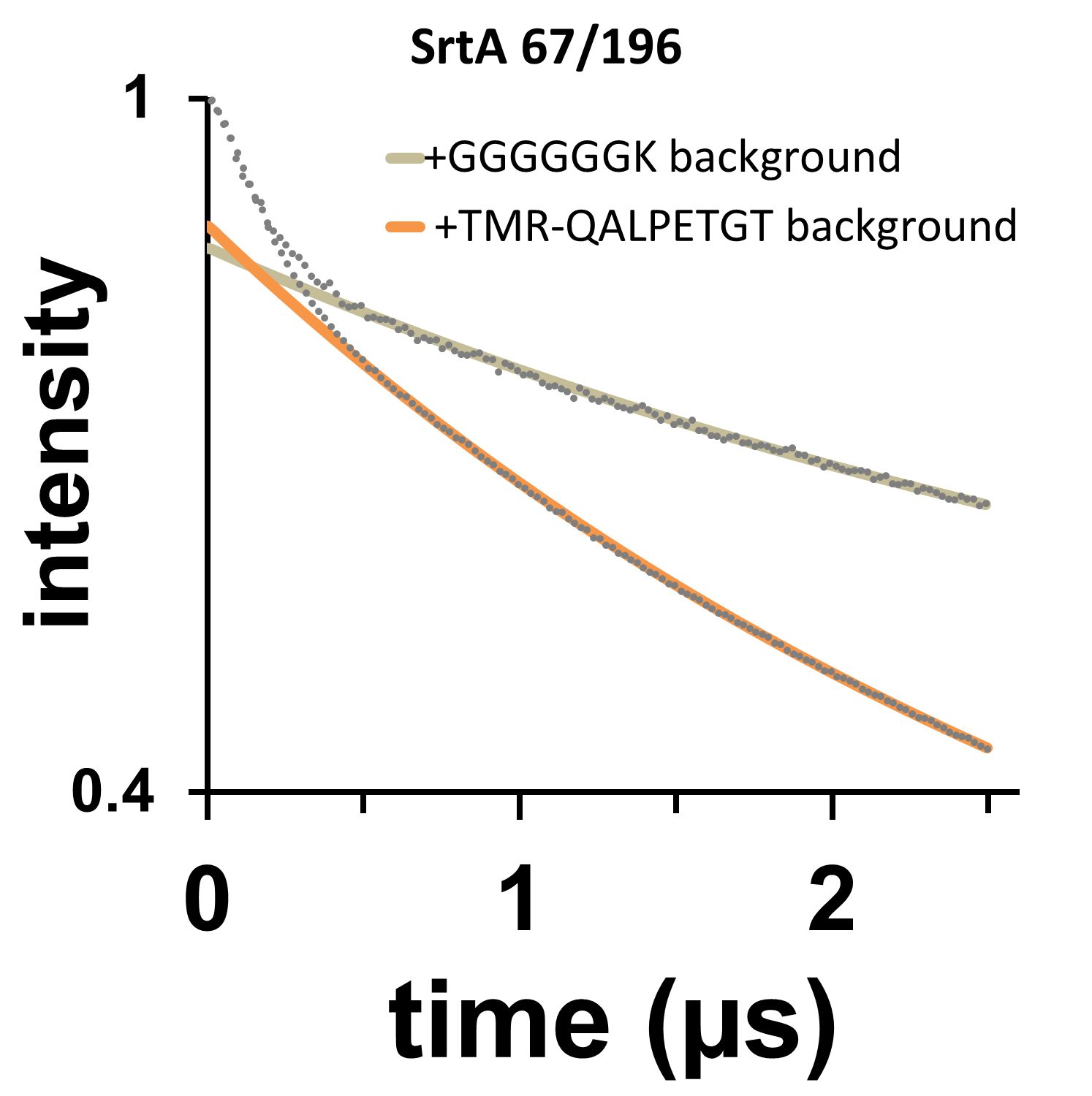
**

**Fig D in S1 File**

**
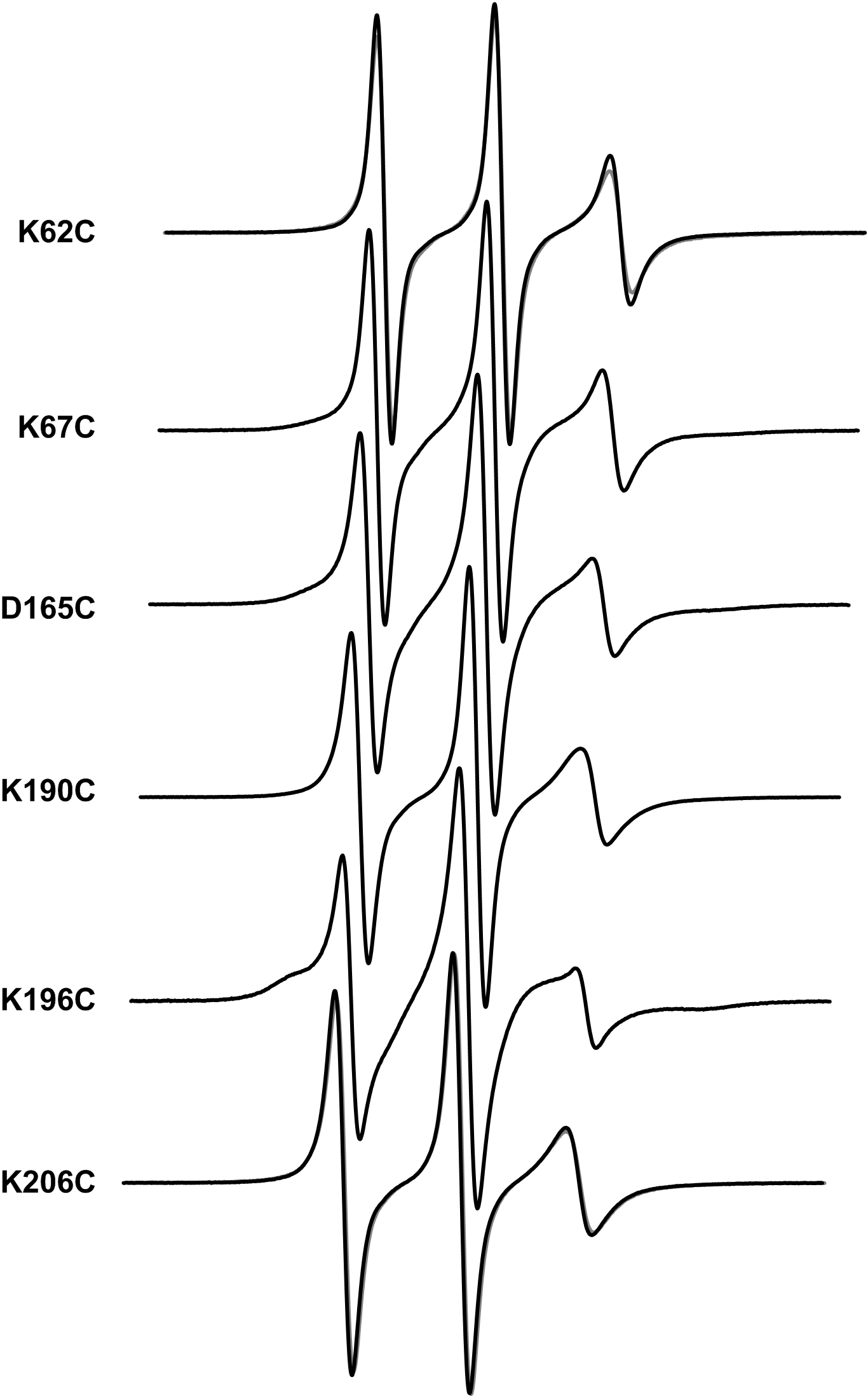
**

**Fig E in S1 File**

Fluorescence polarization Assay: Wild type SrtA

Number of Replicates: 5

**Parameters**

Value ±Std. Error 95% Conf. Interval

Vmax 0.1523 9.433e-3 0.1323 to 0.1723

Km 0.1540 3.689e-2 7.582e-2 to 0.2322


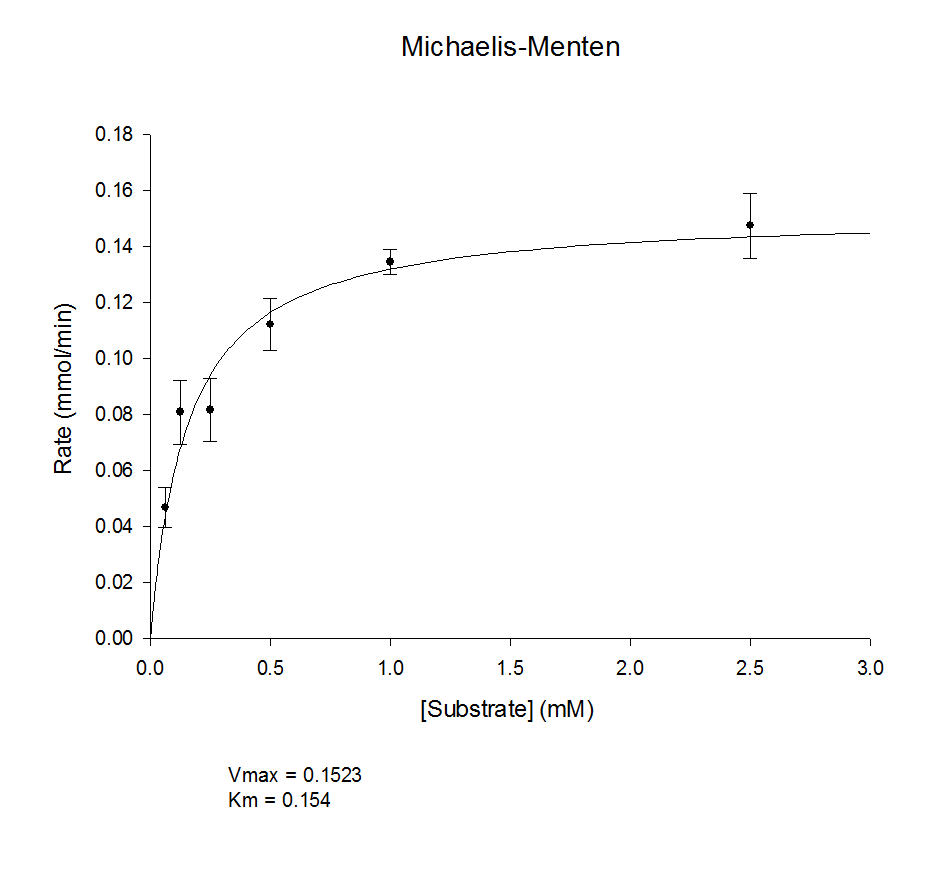

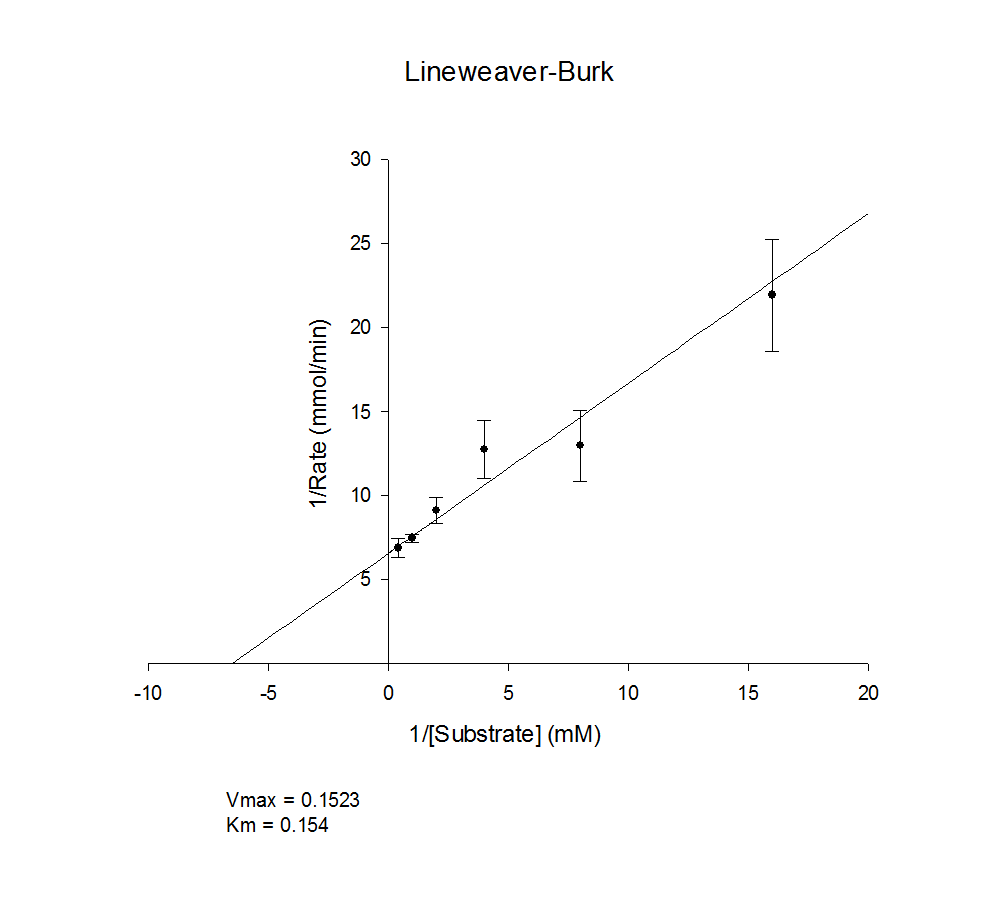


| Experiment 1 |  | | 0 | | 10 | | 20 | | 30 | | mins | |  |
| --- | --- | --- | --- | --- | --- | --- | --- | --- | --- | --- | --- | --- | --- |
|  | 0 G-biotin - sortase | | 14.775 | | 14.395 | | 14.831 | | 14.827 | |  | |  |
|  | 0 G-biotin + sortase | | 15.464 | | 15.232 | | 15.032 | | 14.988 | |  | |  |
|  | 125uM G-biotin | | 15.345 | | 16.24 | | 17.077 | | 16.462 | |  | |  |
|  | 250uM G-biotin | | 15.018 | | 16.263 | | 16.917 | | 17.67 | |  | |  |
|  | 500uM G-biotin | | 15.708 | | 16.82 | | 17.929 | | 18.814 | |  | |  |
|  | 1mM G-biotin | | 15.614 | | 16.921 | | 18.182 | | 19.431 | |  | |  |
|  |  | |  | |  | |  | |  | |  | |  |
| Experiment 2 |  | | 0 | | 10 | | 20 | | 30 | | mins | |  |
|  | 0 G-biotin - sortase | | 14.775 | | 17.735 | | 17.657 | | 17.958 | |  | |  |
|  | 0 G-biotin + sortase | | 15.46 | | 18.451 | | 18.412 | | 18.053 | |  | |  |
|  | 125uM G-biotin | | 15.146 | | 17.974 | | 19.911 | | 20.072 | |  | |  |
|  | 500uM G-biotin | | 17.114 | | 20.74 | | 20.757 | | 22.718 | |  | |  |
|  | 2.5mM G-biotin | | 16.828 | | 20.768 | | 21.881 | | 23.191 | |  | |  |
|  |  | |  | |  | |  | |  | |  | |  |
| Experiment 3 |  | | 0 | | 10 | | 20 | | 30 | | mins | |  |
|  | 0 G-biotin - sortase | | 14.775 | | 14.638 | | 14.738 | | 15.905 | |  | |  |
|  | 0 G-biotin + sortase | | 15.124 | | 14.15 | | 14.027 | | 15.975 | |  | |  |
|  | 62.5uM G-biotin | | 14.431 | | 14.124 | | 15.534 | | 16.563 | |  | |  |
|  | 500uM G-biotin | | 14.83 | | 14.861 | | 14.951 | | 19.074 | |  | |  |
|  | 1mM G-biotin | | 14.812 | | 14.879 | | 16.201 | | 19.463 | |  | |  |
|  | 2.5mM G-biotin | | 13.886 | | 14.784 | | 16.107 | | 19.438 | |  | |  |
|  |  | |  | |  | |  | |  | |  | |  |
| Experiment 4 |  | | 0 | | 10 | | 20 | | 30 | | mins | |  |
|  | 0 G-biotin - sortase | | 14.775 | | 14.897 | | 15.38 | | 15.301 | |  | |  |
|  | 0 G-biotin + sortase | | 15.65 | | 15.64 | | 15.613 | | 15.308 | |  | |  |
|  | 62.5uM G-biotin | | 15.707 | | 16.478 | | 15.585 | | 16.973 | |  | |  |
|  | 125uM G-biotin | | 15.123 | | 15.216 | | 15.577 | | 17.797 | |  | |  |
|  | 250uM G-biotin | | 15.759 | | 16.417 | | 16.67 | | 17.957 | |  | |  |
|  | 1mM G-biotin | | 15.447 | | 16.709 | | 17.193 | | 19.318 | |  | |  |
|  | 2.5mM G-biotin | | 15.576 | | 16.088 | | 18.518 | | 19.824 | |  | |  |
|  |  | |  | |  | |  | |  | |  | |  |
| Experiment 5 |  | | 0 | | 10 | | 20 | | 30 | | mins | |  |
|  | 0 G-biotin - sortase | | 14.775 | | 13.055 | | 11.137 | | 15.173 | |  | |  |
|  | 0 G-biotin + sortase | | 13.544 | | 14.073 | | 13.708 | | 15.366 | |  | |  |
|  | 250uM G-biotin | | 14.17 | | 16.019 | | 16.511 | | 17.818 | |  | |  |
|  | 500uM G-biotin | | 13.046 | | 14.509 | | 16.341 | | 18.579 | |  | |  |
|  | |  | |  | |  | |  | |  | |  | |

Fluorescence polarization Assay: P94H/A104T/E105D/G167E/Q172H Sortase A

Number of Replicates: 3

**Parameters**

Value ±Std. Error 95% Conf. Interval

Vmax 0.2271 5.091e-3 0.2161 to 0.2381

Km 0.3113 2.553e-2 0.2561 to 0.3664


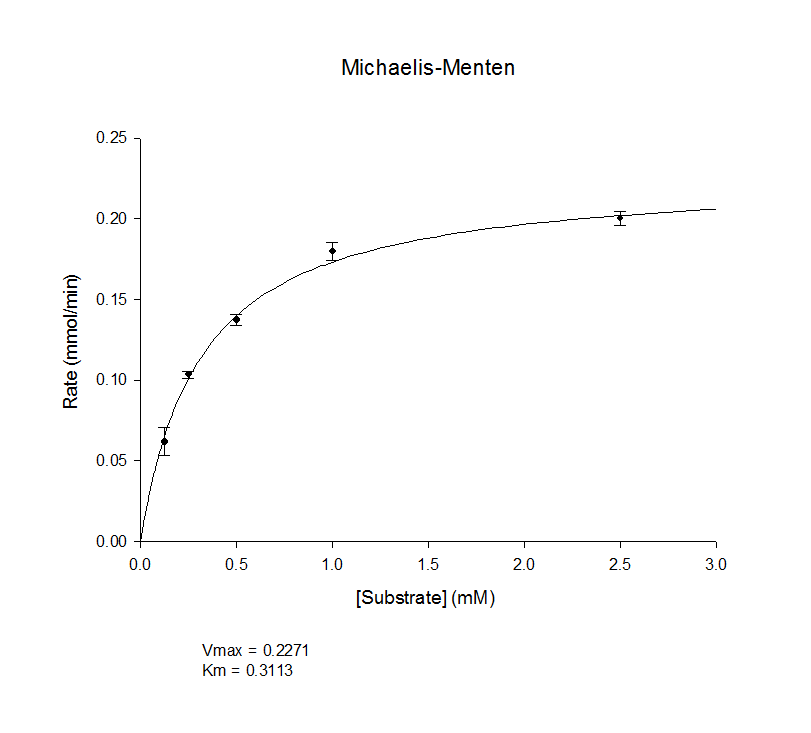

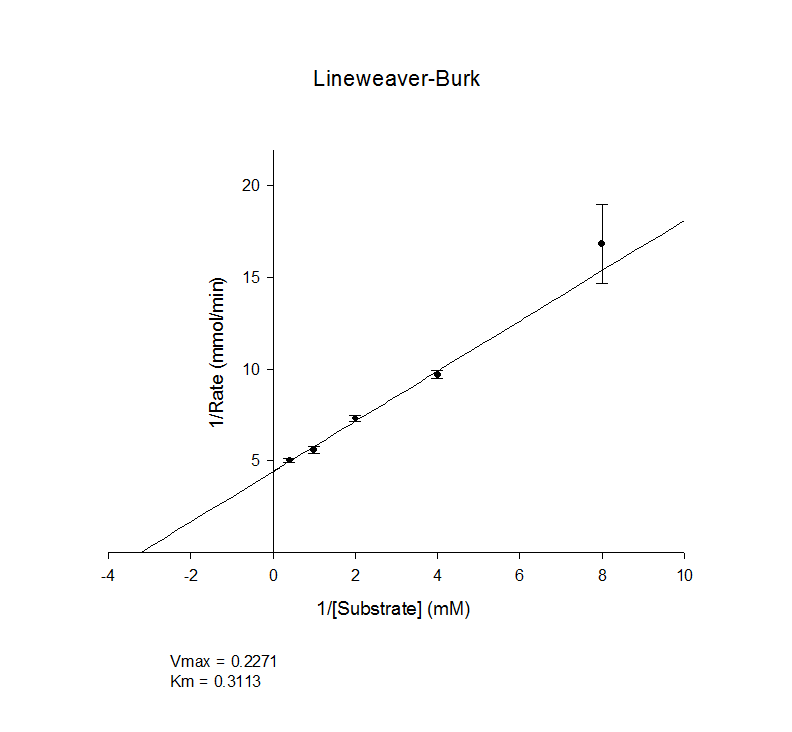


| Experiment 1 |  | | 0 | | 5 | 10 | | 20 | | 30 | mins | |  |  |  |
| --- | --- | --- | --- | --- | --- | --- | --- | --- | --- | --- | --- | --- | --- | --- | --- |
|  | 0 G-biotin - sortase | | 14.775 | | 13.412 | 13.64 | | 13.217 | | 14.506 |  | |  |  |  |
|  | 0 G-biotin + sortase | | 14.57 | | 13.835 | 14.045 | | 13.992 | | 14.692 |  | |  |  |  |
|  | 125uM G-biotin | | 14.397 | | 14.871 | 14.584 | | 15.958 | | 16.269 |  | |  |  |  |
|  | 250uM G-biotin | | 14.022 | | 15.181 | 15.014 | | 16.64 | | 17.543 |  | |  |  |  |
|  | 500uM G-biotin | | 14.718 | | 14.794 | 15.657 | | 16.876 | | 18.826 |  | |  |  |  |
|  | 1mM G-biotin | | 14.47 | | 14.694 | 15.509 | | 17.189 | | 19.708 |  | |  |  |  |
|  | 2.5mM G-biotin | | 14.27 | | 14.37 | 15.633 | | 17.278 | | 20.179 |  | |  |  |  |
|  |  | |  | |  |  | |  | |  |  | |  |  |  |
|  |  | |  | |  |  | |  | |  |  | |  |  |  |
| Experiment 2 |  | | 0 | | 5 | 10 | | 20 | | 30 | mins | |  |  |  |
|  | 0 G-biotin - sortase | | 14.775 | | 13.79 | 13.736 | | 13.716 | | 13.813 |  | |  |  |  |
|  | 0 G-biotin + sortase | | 14.305 | | 14.582 | 14.34 | | 14.571 | | 14.769 |  | |  |  |  |
|  | 125uM G-biotin | | 13.611 | | 15.557 | 16.086 | | 16.736 | | 16.793 |  | |  |  |  |
|  | 250uM G-biotin | | 14.206 | | 16.146 | 16.275 | | 17.801 | | 18.08 |  | |  |  |  |
|  | 500uM G-biotin | | 14.269 | | 15.963 | 16.78 | | 17.785 | | 19.364 |  | |  |  |  |
|  | 1mM G-biotin | | 14.629 | | 15.122 | 16.744 | | 17.895 | | 20.661 |  | |  |  |  |
|  | 2.5mM G-biotin | | 14.392 | | 15.377 | 16.569 | | 18.24 | | 21.007 |  | |  |  |  |
|  |  | |  | |  |  | |  | |  |  | |  |  |  |
|  |  | |  | |  |  | |  | |  |  | |  |  |  |
| Experiment 3 |  | | 0 | | 5 | 10 | | 20 | | 30 | mins | |  |  |  |
|  | 0 G-biotin - sortase | | 14.775 | | 13.444 | 13.789 | | 14.044 | | 15.648 |  | |  |  |  |
|  | 0 G-biotin + sortase | | 14.241 | | 14.308 | 14.44 | | 13.75 | | 15.743 |  | |  |  |  |
|  | 125uM G-biotin | | 14.511 | | 15.24 | 15.544 | | 15.575 | | 17.535 |  | |  |  |  |
|  | 250uM G-biotin | | 15.101 | | 15.257 | 16.46 | | 16.755 | | 19.343 |  | |  |  |  |
|  | 500uM G-biotin | | 15.291 | | 14.646 | 16.717 | | 17.31 | | 20.339 |  | |  |  |  |
|  | 1mM G-biotin | | 14.38 | | 15.527 | 16.349 | | 18.38 | | 21.171 |  | |  |  |  |
|  | 2.5mM G-biotin | | 13.929 | | 15.604 | 16.169 | | 18.432 | | 21.524 |  | |  |  |  |
|  | |  | |  | | |  | |  | | |  | |  |  |

Continuous FRET Assay: Wild type raw data

Substrate: EDANS-QALPETGEE-DABCYL

Fluorescence versus time over 8 substrate concentrations

| Seconds | 0mM | 0.1mM | 0.2mM | 0.5mM | 1.0mM | 2.0mM | 5.0mM | 10.0mM |
| --- | --- | --- | --- | --- | --- | --- | --- | --- |
| 0 | 660.25 | 890 | 1355.5 | 2240.8 | 2649.5 | 3920.8 | 5101 | 6786 |
| 300 | 645.5 | 954.25 | 1444.3 | 2437 | 2891 | 4319.3 | 5815.3 | 7887.5 |
| 600 | 632.25 | 1020.3 | 1564.3 | 2687.8 | 3232 | 4746.5 | 6598.5 | 8974.3 |
| 900 | 641.75 | 1092.3 | 1686.8 | 2951.8 | 3505 | 5246.3 | 7447.8 | 10206 |
| 1200 | 613 | 1156.5 | 1832.3 | 3243.3 | 3870 | 5783.8 | 8324.8 | 11509 |
| 1500 | 569.25 | 1226.8 | 1979.3 | 3527 | 4203 | 6305.8 | 9286.5 | 12887 |
| 1800 | 562.75 | 1325.8 | 2137.8 | 3841.8 | 4606.3 | 6890.5 | 10242 | 14360 |
| 2100 | 547.25 | 1441 | 2294.5 | 4186.5 | 5018 | 7524.3 | 11380 | 15921 |
| 2400 | 534.25 | 1541.3 | 2487 | 4524.3 | 5448.3 | 8149.8 | 12479 | 17615 |
| 2700 | 538.5 | 1610.8 | 2644 | 4858.3 | 5849.8 | 8793.3 | 13696 | 19399 |
| 3000 | 531.25 | 1700.8 | 2799 | 5202.8 | 6292 | 9529.3 | 14905 | 21073 |
| 3300 | 533 | 1790 | 2960.8 | 5539.5 | 6767 | 10205 | 16222 | 22892 |
| 3600 | 523 | 1891.3 | 3121.5 | 5901.8 | 7157.8 | 10784 | 17615 | 24558 |
| 3900 | 515 | 1979.8 | 3327 | 6206.5 | 7596.8 | 11522 | 18821 | 26152 |
| 4200 | 531 | 2058.5 | 3472.8 | 6478.5 | 7932 | 12054 | 20036 | 27461 |

After background correction and linear fitting of fluorescence intensity against time:

| [Substrate] | (Velocity) |
| --- | --- |
| 0 | 0 |
| 0.1 | 0.731664629 |
| 0.2 | 1.177851836 |
| 0.5 | 2.123735361 |
| 1 | 2.564316348 |
| 2 | 3.853262603 |
| 5 | 5.921572221 |
| 10 | 8.124889923 |

Continuous FRET Assay: P94H/A104T/E105D/G167E/Q172H Sortase A

Substrate: EDANS-QALPETGEE-DABCYL

Fluorescence versus time over 8 substrate concentrations

| Raw Data |  |  |  |  |  |  |  |  |
| --- | --- | --- | --- | --- | --- | --- | --- | --- |
| Seconds | 0mM | 0.1mM | 0.2mM | 0.5mM | 1.0mM | 2.0mM | 5.0mM | 10.0mM |
| 0 | 3664.3 | 5966.5 | 8256.5 | 9169 | 11831 | 15548 | 24706 | 33295 |
| 300 | 3733 | 6073.8 | 8068 | 9574.3 | 12490 | 16745 | 26577 | 35612 |
| 600 | 3665.3 | 6227 | 8477 | 9820.8 | 13880 | 18009 | 29031 | 38794 |
| 900 | 3601 | 6477.8 | 8557.5 | 10428 | 14628 | 20035 | 31724 | 41073 |
| 1200 | 3602.5 | 6420.3 | 8962.3 | 11022 | 15582 | 21354 | 33798 | 43570 |
| 1500 | 3582 | 6636.8 | 9010 | 11638 | 16389 | 22746 | 36410 | 45794 |
| 1800 | 3708.5 | 6923 | 9522.8 | 11681 | 17155 | 23377 | 39769 | 49353 |
| 2100 | 3759.5 | 7210 | 9958.3 | 12819 | 18404 | 25592 | 41827 | 53031 |
| 2400 | 3547 | 7206.3 | 10269 | 14195 | 19513 | 27456 | 45612 | 55476 |
| 2700 | 3705.3 | 7429.3 | 10861 | 14458 | 20794 | 28521 | 48231 | 58070 |
| 3000 | 3740 | 7621 | 11023 | 14962 | 22087 | 30798 | 51276 | 60534 |
| 3300 | 3724 | 8233.8 | 11297 | 15668 | 23554 | 33526 | 53404 | 62149 |
| 3600 | 3762.5 | 8359 | 12114 | 16509 | 25194 | 34531 | 56884 | 62828 |
| 3900 | 4016.8 | 8582.3 | 12507 | 17383 | 26303 | 36882 | 59312 | 63220 |
| 4200 | 3936.8 | 9089.8 | 13025 | 18592 | 27803 | 39326 | 61114 | 63370 |
|  |  |  |  |  |  |  |  |  |

After background correction and linear fitting of fluorescence intensity against time:

| [Substrate] | Velocity |
| --- | --- |
| 0 | 0 |
| 0.1 | 5.399099438 |
| 0.2 | 7.385282646 |
| 0.5 | 9.188770763 |
| 1 | 12.86922036 |
| 2 | 17.5156355 |
| 5 | 28.29433923 |
| 10 | 35.83113642 |
